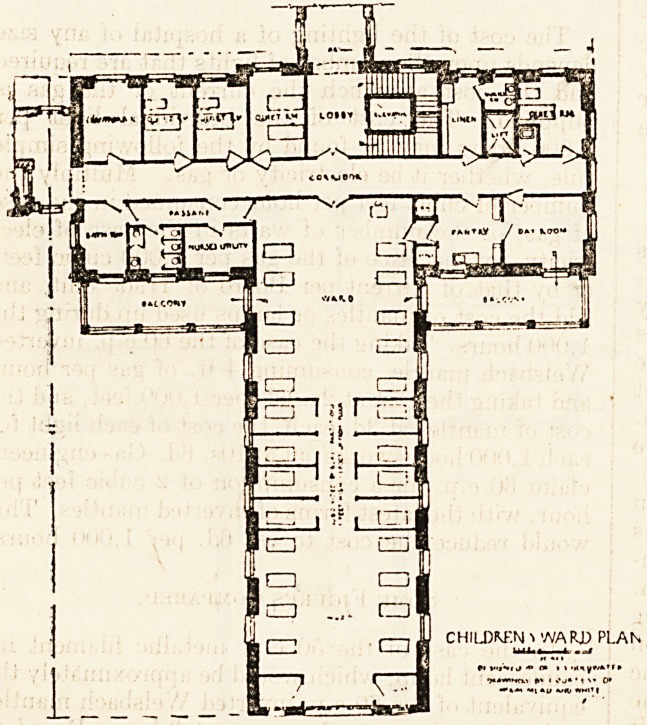# Notes on Hospital Planning

**Published:** 1911-02-04

**Authors:** S. S. Goldwater

**Affiliations:** Superintendent Mount Sinai Hospital, N.Y., Consulting Supervisor of Construction to Bellevue Hospital, Stamford Hospital, etc.


### SPECIAL INSTITUTIONAL ARTICLE.

### NOTES ON HOSPITAL PLANNING.

By S. S. GOLDWATER, M.D., Superintendent Mount Sinai Hospital, N.Y., Consulting Supervisor of Construction to Bellevue Hospital, Stamford Hospital, etc.

(Abstract of Paper read before Congress of American Hospitals Association.)

An acceptable plan for the construction of ward buildings of many stories in crowded cities has long been needed. Such a plan must satisfy the requirements of convenient administration, and must comply in all essentials with the demands of hygiene, even under the hard conditions of a restricted site and of possibly unfavourable surroundings.

It is assumed that economic necessity compels us, and will compel us indefinitely, to continue to house a majority of hospital patients in large wards. Those who are opposed to large wards and who propose to provide for each patient the particular environment best suited to his condition and needs are no doubt correct in theory. A private room with a porch and a garden; a private nurse on day duty and another on night duty; a skilled medical officer, not too much distracted with administrative duties or with the care of other patients—all these combined represent a kind of hospital organisation which is greatly to be desired, because in the long run it would yield the best results in the treatment of patients acutely ill.

### The Subdivided Ward.

The folly of subdividing wards into single rooms, while there is a lack of means to increase substantially the number of nurses, has been demonstrated to the satisfaction of more than one hospital superintendent, and to the serious discomfiture of patients in wards subdivided and understaffed.

Nevertheless the necessity of a partial classification of patients within the typical medical or surgical ward group must be recognised, even if a complete and perfect classification is at present unattainable; this necessity is recognised in the accompanying ward plan, as it is in all ward plans which provide, among the appendages, a lounging and dining-room for convalescents, an airing balcony or balconies, and one or more "recovery," isolating, or "quiet" rooms.

### Problems of Ward Planning.

The problem in ward planning is to bring together all these helps to good nursing and proper care in such a manner as to facilitate their supervision by the limited number of nurses at present available, and at the same time to avoid hemming in the ward itself in such a way as to interfere materially with its supply of light and air.

The modern hospital must be able to place its patients out of doors, whether in gardens or roof-wards, or on loggias or balconies. Now since in crowded cities we cannot have gardens, and since roof-wards can only be utilised for a relatively small number of patients, the principal wards must have balconies; and these must be so placed as to be sun-warmed in winter, must be accessible for both bed-patients and convalescents, must lend themselves readily to constant supervision, and must be so arranged as neither to disfigure the building nor greatly to darken the wards. Besides this, the balconies must not be too close to the street.

It is essential also, on account of the rapidly increasing hospital needs of urban communities, that the ward plan shall be one which, if utilised at first for the construction of a four or five storey building, will permit us to superimpose new wards upon the old ones without detriment to the latter; and it is essential so to locate our ward buildings with relation to the other buildings of the hospital group that these other buildings, in their turn, may be increased in height and doubled in capacity, if necessary, without any signal alteration in the hygienic character of the wards.

TYPICAL WARD PLAN

SCALE

DESIGNED BY DR. S. S. GOLDWATER

DRAWINGS BY COURTESY OF [illegible] MEAD AND WHITE

This is not all that is required by the conditions of our problem. If the ward buildings, fronting south, can be so placed as to face a park or an open lot, well and good; but inasmuch as such sites are not always available, and since empty lots do not always remain unoccupied, our plan must be one which will not lose much of its virtue if open ground on the opposite or south side of the street is not available, or if such open ground, present at the time of the construction of the hospital, is subsequently covered with buildings.

### The T-shaped Ward.

The use of the T-shaped ward building enables us to construct a full-sized ward of thirty-one beds (five of which are in "separation" rooms) within a space extending only 120 feet from north to south, or a ward of twenty-six beds within a space extending

106 feet from north to south. If we leave to the north of this an air-zone of 30 feet in the one case, or 44 feet in the other, there will be available for administration and service buildings 50 feet along the line of the street which forms the northerly margin of a block extending 200 feet from north to south. If the ward appendages and main service corridor were extended in the axis of the ward (as in the case of the typical pavilion hospitals of Germany and Great Britain), 150 to 170 feet would be required from north to south for the ward building alone, and the remainder of the 200-foot site would be of little or no use.

A study of the group plans shows that as much as

60 per cent. of the total ground area of a site 200 by 200 feet, 200 by 350 feet, 200 by 500 feet, etc., may be occupied by buildings with satisfactory results.

### Balconies and Observation.

The wards are well exposed on two long sides and one short side, east, west, and south; the balconies or loggias are ample in capacity and have the decided advantage (in this climate, at least) of southern exposure. They do not to any appreciable extent darken the wards, and they are under the eye of the nurses in the ward; furthermore, they are so subdivided that convalescent patients may amuse themselves without restraint on one balcony, while very sick bed-patients are obtaining the benefits of fresh-air treatment, in undisturbed quiet, on the other. Each balcony is directly visible from one of the principal service rooms, namely, the pantry or the sink-room. The balconies are set back at a comfortable distance from the street.

### Corridors and Ventilation.

The balcony, day-room, lavatories, and water-closets designed for the use of convalescent patients are grouped about one end of the main corridor; the isolation of the very sick takes place at the opposite end of the corridor, convenient to the principal service rooms, and entirely out of the range of observation of the convalescent patients and their friends.

The stairway and elevator lobby is isolated and yet occupies an especially favourable location, directly opposite the main entrance to the ward. Visitors approaching the ward do not pass through a long service corridor, but find their way immediately to their proper destination.

The principal corridor is arranged to serve as a true cross-ventilating corridor.

The horizontal arm of the " T," running east and west, can be lengthened, and the vertical arm shortened, if desired, for the purpose of increasing the number of separation rooms and of diminishing the number of patients in the open ward.

A special modification of the typical ward plan, to meet the altered requirements of a children's service, is included among the sketches submitted. Features of this plan are the observation windows permitting the control of the children's water-closets from the nurses' utility-room; the rooms for isolated cases or for babies and wet-nurses; the glass " boxes " for semi-isolation within the large ward; the larger bathroom, to accommodate bath tub and slab.

### The Group Plan.

Bridges may be carried from the ward buildings to the north, east, or west, without detriment to the wards. In a group plan including two ward buildings, a bridge to the east or west would give convenient access to a central administration building. In a group plan including but one ward building, a bridge to the north would communicate with an administration building facing the northerly street; in a larger group plan, bridges to the north would communicate, according to the details of the general scheme, with an administration building, kitchen and laundry building, pathological laboratory, operating pavilion, out-patient department, or with buildings used for any variety or combination of the purposes named. In the larger and more complete group plans a separate out-patient building, not too high, would be placed at the south-east or south-west corner of the block, and would be balanced by a private patients' pavilion at the opposite corner, leaving the ward buildings well exposed.

### The Scheme's Originality.

The essential feature of the scheme herewith presented, in which it differs from any published or applied ward plan known to the writer, is the combination of ward and balcony in a T-shaped plan, which, under the common conditions of hospital construction in crowded cities, seems to offer advantages not otherwise attainable.

## Figures and Tables

**Figure f1:**
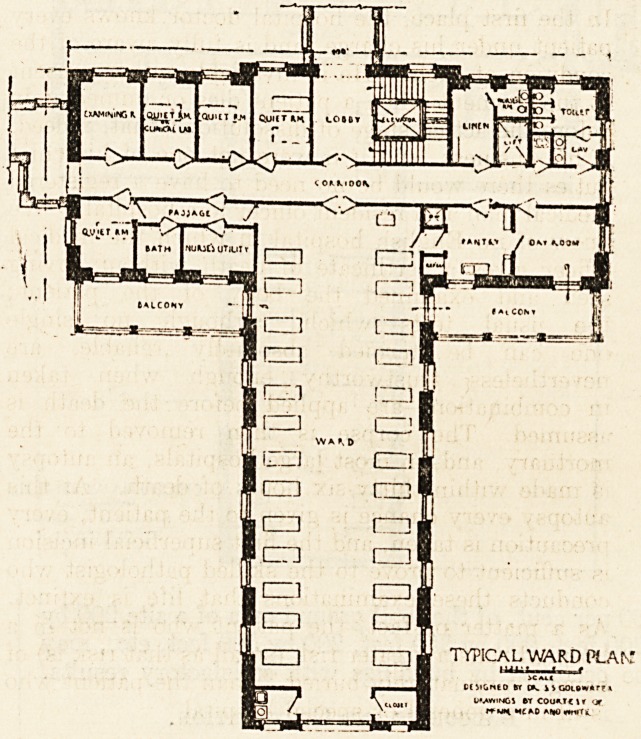


**Figure f2:**